# Identification of shared molecular mechanisms and diagnostic biomarkers between heart failure and idiopathic pulmonary fibrosis

**DOI:** 10.1016/j.heliyon.2024.e30086

**Published:** 2024-04-20

**Authors:** Peng Zhang, Lou Geng, Kandi Zhang, Dongsheng Liu, Meng Wei, Zheyi Jiang, Yihua Lu, Tiantian Zhang, Jie Chen, Junfeng Zhang

**Affiliations:** a Department of Cardiology, Shanghai Ninth People's Hospital, Shanghai Jiao Tong University School of Medicine, Shanghai, China; b Department of Hematology, Institute of Hematology, Changhai Hospital, Naval Medical University, Shanghai, China

**Keywords:** Heart failure, Idiopathic pulmonary fibrosis, Bioinformatics, WGCNA, SVM-RFE, Diagnostic biomarkers

## Abstract

**Background:**

Heart failure (HF) and idiopathic pulmonary fibrosis (IPF) are global public health concerns. The relationship between HF and IPF is widely acknowledged. However, the interaction mechanisms between these two diseases remain unclear, and early diagnosis is particularly difficult. Through the integration of bioinformatics and machine learning, our work aims to investigate common gene features, putative molecular causes, and prospective diagnostic indicators of IPF and HF.

**Methods:**

The Gene Expression Omnibus (GEO) database provided the RNA-seq datasets for HF and IPF. Utilizing a weighted gene co-expression network analysis (WGCNA), possible genes linked to HF and IPF were found. The Kyoto Encyclopedia of Genes and Genomes (KEGG) and Gene Ontology (GO) were then employed to analyze the genes that were shared by HF and IPF. Using the cytoHubba and iRegulon algorithms, a competitive endogenous RNA (ceRNA) network was built based on seven basic diagnostic indicators. Additionally, hub genes were identified using machine learning approaches. External datasets were used to validate the findings. Lastly, the association between the number of immune cells in tissues and the discovered genes was estimated using the CIBERSORT method.

**Results:**

In total, 63 shared genes were identified between HF- and IPF-related modules using WGCNA. Extracellular matrix (ECM)/structure organization, ECM-receptor interactions, focal, and protein digestion and absorption, were shown to be the most enrichment categories in GO and KEGG enrichment analysis of common genes. Furthermore, a total of seven fundamental genes, including *COL1A1*, *COL3A1*, *THBS2*, *CCND1*, *ASPN*, *FAP*, and *S100A12*, were recognized as pivotal genes implicated in the shared pathophysiological pathways of HF and IPF, and TCF12 may be the most important regulatory transcription factor. Two characteristic molecules, *CCND1* and *NAP1L3*, were selected as potential diagnostic markers for HF and IPF, respectively, using a support vector machine-recursive feature elimination (SVM-RFE) model. Furthermore, the development of diseases and diagnostic markers may be associated with immune cells at varying degrees.

**Conclusions:**

This study demonstrated that ECM/structure organisation, ECM-receptor interaction, focal adhesion, and protein digestion and absorption, are common pathogeneses of IPF and HF. Additionally, *CCND1* and *NAP1L3* were identified as potential diagnostic biomarkers for both HF and IPF. The results of our study contribute to the comprehension of the co-pathogenesis of HF and IPF at the genetic level and offer potential biological indicators for the early detection of both conditions.

## 1 Introduction

HF is generally defined as systolic or diastolic dysfunction of the heart that does not amply pump blood out of the heart, causing venous congestion and inadequate blood supply to the arterial system [1]. Per the latest European guidelines, HF is a clinical illness characterised by an irregularity in the heart's structure and function which leads to insufficient cardiac output and increased intracardiac pressure [2]. HF is considered a global pandemic and its incidence has increased in recent years [3]. Statistics have revealed that 26 million people worldwide have been diagnosed with HF, of whom approximately 54 % die within four years of diagnosis [4]. Patients with HF have a high death and morbidity rate, particularly those aged 65 and older, which places a heavy burden on global healthcare spending [3,5]. Until now, the specific molecular mechanism of HF remains unclear; therefore, study is urgently required to enhance our understanding of its biological mechanism. Idiopathic pulmonary fibrosis (IPF) is a long-lasting and advancing lung illness that involves the development of inexplicable scarring and substantial remodeling of the lungs [6]. Although the prevalence of IPF varies globally, studies have shown that its estimated prevalence ranges between 3 and 25 in Europe, 6–45 in Asia, and 24–30 per 100, 000 persons in North America [7].

More seriously, owing to rapid disease progression, people diagnosed with IPF often have a median survival of only two-five years [6,8]. Importantly, IPF is often accompanied by cardiovascular diseases, including HF [[9], [10], [11]]. For instance, an investigation revealed that HF accounted for 16 % of the deaths in 326 patients with IPF [12]. A separate study illustrated that the occurrence of HF is notably less common in people without IPF compared to those with IPF. Furthermore, HF is an independent prognostic factor for patients with IPF, suggesting that screening and treating HF can be advantageous for the clinical care of IPF patients [13]. Although there is clear evidence of a link between HF and IPF, their common characteristics based on gene regulation mechanisms remain to be elucidated.

Therefore, it is imperative to find biomarkers that are associated with the onset and progression of HF and IPF in order to accurately diagnose and treat these conditions. Advancements in sequencing technology and bioinformatics have enabled us to uncover the co-pathogenesis of disease-disease interactions at the genetic level. This study aimed to explore common genetic features, underlying molecular mechanisms, and potential diagnostic indicators of both diseases by integrating machine learning techniques and bioinformatics. In our study, we chose the GSE29819 and GSE24206 datasets because they were relevant to our research question and their sample sizes were over 15, making them suitable for WGCNA analysis.

## 2 Methods

### 2.1 Dataset download and processing

The initial gene expression profile data were acquired from the GEO database [14]. An inquiry was performed in the GEO database utilizing the terms "heart failure" and "idiopathic pulmonary fibrosis" to identify RNA-seq profiles. Two GEO datasets, GSE29819 and GSE24206, were obtained for training purposes (training set) with a sample size larger than the recommendation for WGCNA (N > 15). The gene expression data from RNA-seq was normalized using quantile normalization with the "normalizebetweenarrays" function in the "limma" package. Additionally, the data was converted to log2 scale. Table 1 provides comprehensive details on the datasets, including the microarray platform used, the sample groups involved, and the corresponding numbers.

**Table 1:** Basic information of GEO datasets used in the study.

ID	GSE series	Disease	Samples	Sourcetypes	Platform	Group
1	GSE29819	HF	14 DCM samples and 12 normal controls	Heart	GPL570	Training cohort
2	GSE24206	IPF	17 IPF samples and 6 normal controls	Lung	GPL570	Training cohort
3	GSE21610	HF	60 HF samples and 8 normal controls	Heart	GPL570	Validation cohort
4	GSE53845	IPF	40 IPF samples and 8 normal controls	Lung	GPL6480	Validation cohort

HF, heart failure; IPF, idiopathic pulmonary fibrosis; DCM, dilated cardiomyopathy.

### 2.2 WGCNA analysis

The unsupervised method of WGCNA was employed to discover gene modules that have similar expression characteristics across samples [15]. This investigation utilized the "WGCNA" package in R version 4.3.0. The "pick Soft Threshold" function was used to select soft-power parameters within the range of 1–20, based on the criterion of scale-free topology. The chosen values were utilized to build an adjacency matrix. Afterwards, the most suitable β value was identified to turn the correlation matrix into an adjacency matrix, which was then converted into a topological overlap matrix. The genes were grouped together based on the topological overlap matrix using the average-linkage hierarchical clustering approach, with a minimum module size of 50. A heatmap was generated using Spearman's correlation coefficient to evaluate the potential correlations between modules and clinical features, in order to analyze the relationships between the modules. Typically, genes are clustered into different modules, and each module is assigned a colour to facilitate visualisation and analysis. The soft threshold β for the WGCNA was set at 5 for HF and 6 for IPF in this study. The selection of these soft-power values was determined by the criterion of scale-free topology, which guaranteed an ideal structure for the co-expression network. The remaining parameters utilized were as follows: The network type is "unsigned", the minimum module size is 20, the merge cut height is 0.25, and the deep split is 2.

### 2.3 Functional enrichment analysis

The GO consists of three categories: cellular components (CC), biological processes (BP), and molecular function (MF) [16]. The KEGG functions as a database for the systematic examination of gene activities, specifically in relation to gene networks [17]. In order to determine the possible roles of the genes in the major modules, we conducted GO and KEGG pathway analyses. Functional enrichment analysis was performed using the R tool "ClusterProfiler". The top 10 GO terms in each category were visualized using the R package "ggplot2" [18]. The threshold for statistical significance was established at a *p*-value of less than 0.05.

### 2.4 Identification of central genes and regulatory factors

The Cytoscape plugin app CytoHubba offers a user-friendly approach for investigating important nodes in networks of biological molecules and for selecting seven methods to examine key genes in a protein–protein interaction (PPI) network. The Cytoscape plugin iRegulon was utilized to analyze the transcription factors responsible for regulating the marker genes [19]. The parameter values employed included a minimum identity threshold of 0.05 for orthologous genes and a maximum false discovery rate of 0.001 for motif similarity. The result yielded the normalized enrichment score (NES), where higher scores signify increased reliability. Transcription factor and target gene pairings with NES values greater than 5 were chosen.

### 2.5 SVM-RFE analysis

The SVM-RFE is a method that sequentially eliminates features using SVM to discover the best key gene. The SVM-RFE method outperforms both the linear discriminant analysis and mean-squared error approach in accurately detecting relevant features and removing unnecessary features. The application of SVM-RFE for feature selection was executed through a rigorous process of ten-fold cross-validation [20]. This process involves the deletion of feature vectors that are dependent on the “e1071” and “msvmRFE” package in R version 4.3.0 for SVM modelling. Core biomarkers were identified using SVM analysis at the intersection of the WCGNA.

### 2.6 ROC analysis

To validate these findings, the GSE21610 and GSE53845 datasets were acquired from the GEO database. The diagnostic efficacy of these hub genes was evaluated using receiver operating characteristic (ROC) curve analysis. The threshold for statistical significance was established at a *p*-value of less than 0.05.

### 2.7 Analysis of immune infiltration

The CIBERSORT algorithm was employed to perform deconvolution by integrating the annotated genomes of distinct immune cell subpopulations, facilitating the calculation of the relative proportions of 22 immune cell types within the tissues. Spearman's non-parametric correlations were employed to determine the relationship between immune-infiltrated cells and fundamental biomarkers [21].

### 2.8 Statistical analysis

The data analysis was performed using R version 4.3.0 (https://www.rproject.org/), Cytoscape software (version 3.9.1), and the R Bioconductor package. The threshold for statistical significance was established at a *p*-value of less than 0.05.

## 3 Results

### 3.1 Weighted gene Co-expression network analysis

**Fig. 1:**
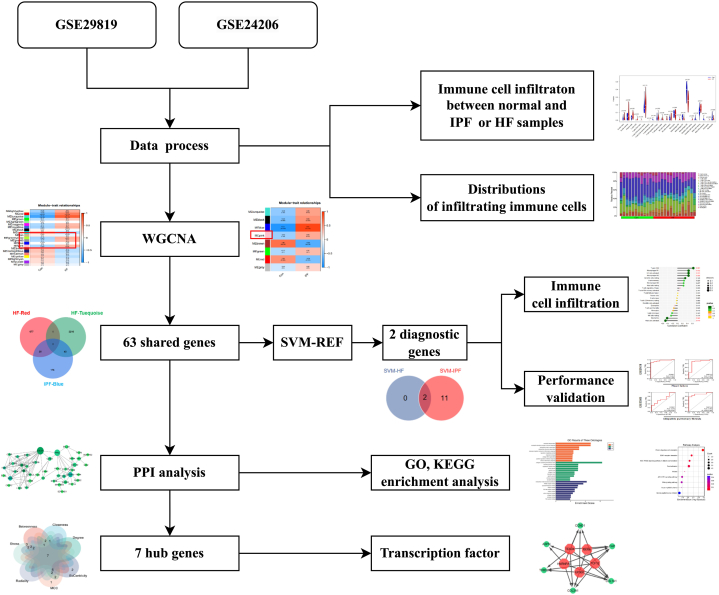
The flowchart of the whole study. Abbreviations: HF, heart failure; IPF, idiopathic pulmonary fibrosis; WGCNA, weighted gene co-expression network analysis; SVM-RFE, support vector machine-recursive feature elimination; GO, gene ontology; KEGG, the kyoto encyclopedia of genes and genomes; PPI, protein‒protein interaction.

**Fig. 2:**
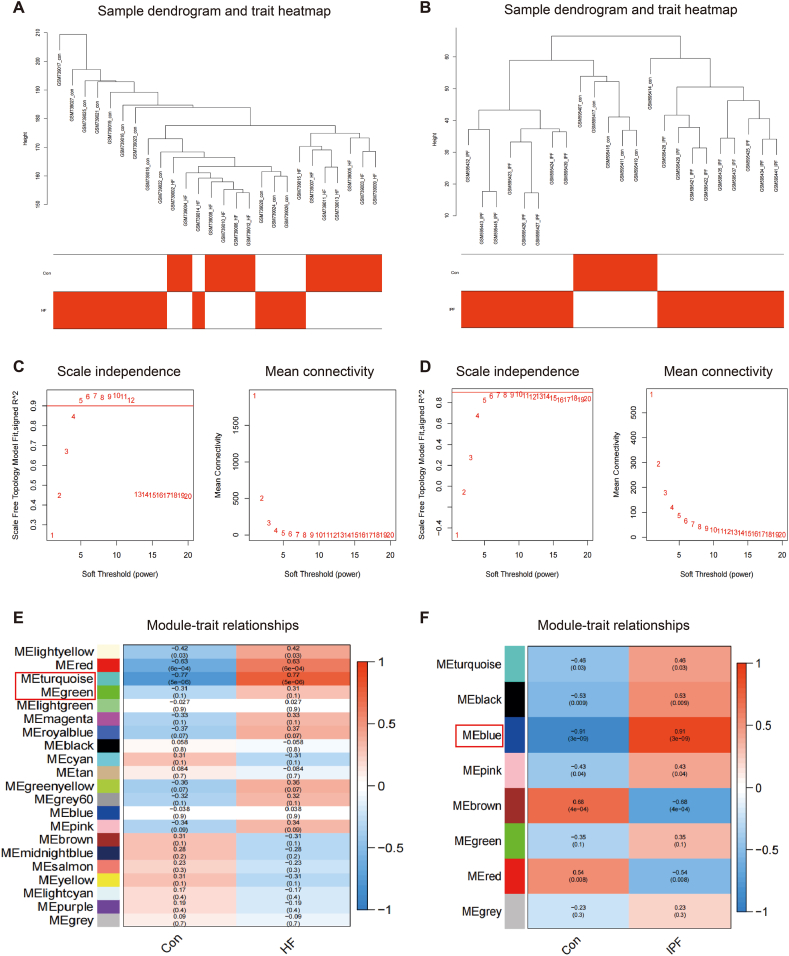
Identification of key modules in HF and IPF samples based on WGCNA analysis. (A) Correlation between modules and genes in GSE29819. (B) Correlation between modules and genes in GSE24206. (C) Determination of soft-thresholding power for GSE29819. (D) Determination of soft-thresholding power for GSE24206. (E) Heatmap of the correlation between module eigengenes and the occurrence of HF. (F) Heatmap of the correlation between module eigengenes and the occurrence of IPF.

The research process is illustrated in Fig. 1. We conducted WGCNA to examine the relationship between clinical data and important genes. Genes that showed a statistically significant difference in expression (*p* < 0.05) were chosen as inputs for WGCNA. All samples were grouped together in the GSE29819 and GSE24206 datasets, and none of them were excluded (Fig. 2A and B). According to the WGCNA technique, a soft-power value of β = 5 was found to be the most effective for GSE29819 (Fig. 2C), whereas a soft-power value of β = 6 was found to be the most effective for GSE24206 (Fig. 2D). In GSE29819, a total of 21 modules were found, while in GSE24206, eight modules were detected. Afterwards, the correlations between the modules and clinical traits were computed. The red (R = 0.63) and turquoise (R = 0.77) modules showed the strongest positive correlations with HF in the GSE29819 dataset (Fig. 2E). In GSE24206, the blue module (R = 0.91) showed the strongest positive correlation with IPF (Fig. 2F). WGCNA helped us screen the most clinically relevant modules in each dataset for further genetic analysis.

### 3.2 Identification and function enrichment of the shared genes

**Fig. 3:**
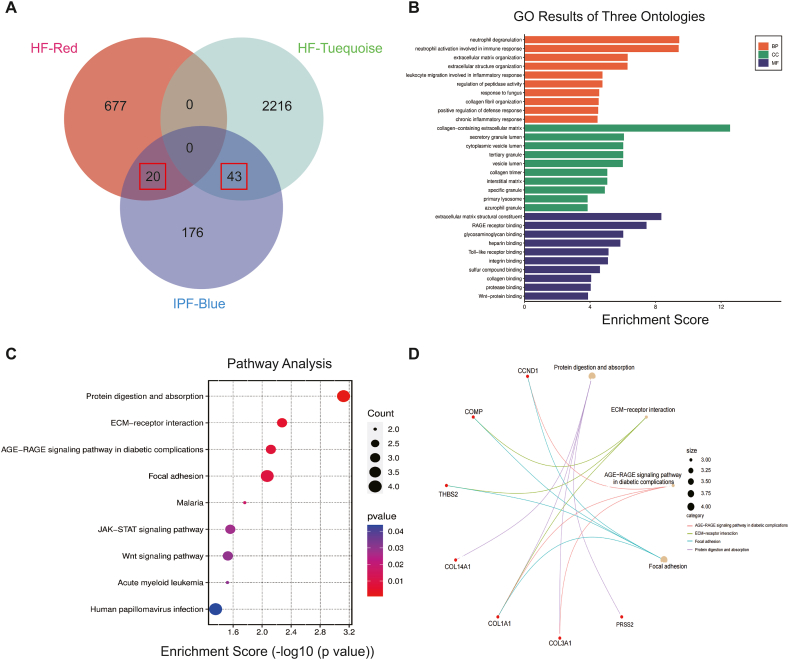
Identification and function enrichment of 63 shared genes for HF and IPF. (A) Identification of shared genes with a Venn diagram. (B) GO enrichment analysis results for 63 shared genes. (C–D) KEGG enrichment analysis results for 63 shared genes.

The most clinically important modules of HF and IPF overlapped by 63 genes (Fig. 3A, Table S1). In order to gain a deeper understanding of the roles of shared genes in HF and IPF, we analysed the biological characteristics of the 63 genes using two gene enrichment analysis methods. GO analysis revealed the MF, BP, and CC associated with the 63 genes. KEGG analysis revealed that major cellular signalling networks were involved in 63 genes. The Gene Ontology (GO) analysis revealed that several biological processes were enriched in the study. These processes include neutrophil degranulation, neutrophil activation associated with the immune response, extracellular matrix (ECM) organization, leukocyte migration associated with the inflammatory response, regulation of peptidase activity, collagen fibril organization, and chronic inflammatory response. The collagen-containing ECM, the lumens of secretory granules and cytoplasmic vesicles, tertiary granules, vesicle lumens, the interstitial matrix, particular granules, primary lysosomes, and azurophil granules were found in higher concentrations in the CC. The extracellular molecular components matrix, the RAGE receptor binding, Toll-like receptor binding, integrin binding, sulphur compound binding, collagen binding, protease binding, and Wnt protein binding, were shown to be more abundant in MF (Fig. 3B). The KEGG analysis revealed that the common genes exhibited enrichment in many signaling pathways, such as protein digestion and absorption, ECM-receptor interaction, JAK-STAT, AGE-RAGE, and Wnt signaling pathway (Fig. 3C and D).

### 3.3 Identification of core genes and transcription factors

**Fig. 4:**
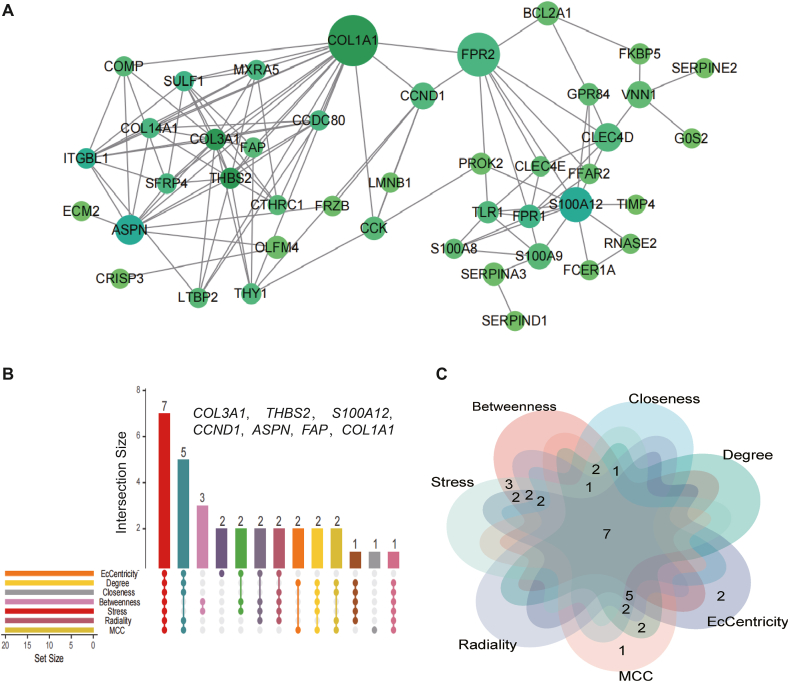
Identification of hub genes in HF and IPF. (A) PPI network of shared genes. (B–C) Upset plot and Venn diagram showing intersected genes obtained by 7 algorithms in PPI analysis.

In order to identify the potential pathogenic genes and underlying mechanisms in HF and IPF, we created a PPI network using common genes. This network was constructed using STRING, which allowed us to investigate the potential relationships. We set a minimum needed interaction score of 0.4 and a PPI enrichment *p*-value of less than 1.0e-16 (Fig. 4A).

**Fig. 5:**
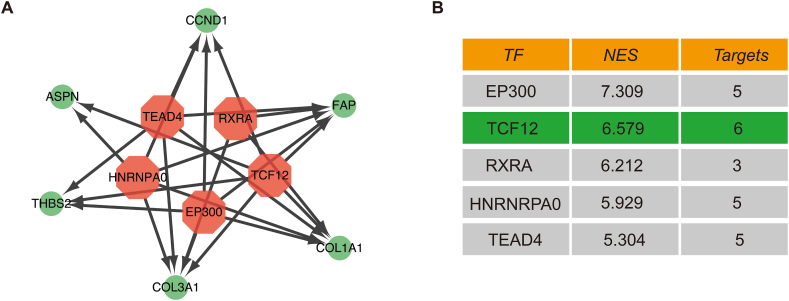
Prediction of TF genes and their interaction network with hub genes. (A) Five transcription factors with an NES score>5 was predicted by iRegulon and visualized by the Cytoscape. We showed regulatory network between transcription factors and targeted genes. B. The NES value of TF genes.

Furthermore, by employing seven methods (Betweenness, Stress, Radiality, Closeness, Degree, EcCentricity, and MCC), we have successfully determined *COL3A1*, *THBS2*, *S100A12*, *CCND1*, *ASPN*, *FAP*, and *COL1A1* as core genes (Fig. 4B and C). The transcriptional regulatory networks of these key genes are depicted in Fig. 5A and B. The transcription factors with a NES score greater than 5 include EP300 (E1A binding protein p300, 7.309), RXRA (Retinoid X Receptor Alpha, 6.212), TCF12 (Transcription Factor 12, 6.579), HNRNPA0 (Heterogeneous Nuclear Ribonucleoprotein A0, 5.929), and TEAD4 (TEA domain transcription factor 4, 5.304). Through the interaction network between protein molecules and the scores of transcription factors, we further screened the most relevant seven genes and one transcription factor among the 63 shared genes.

### 3.4 Identification of Two Shared Hub Genes and their Potential Diagnostic Value utilizing a Machine Learning Algorithm

**Fig. 6:**
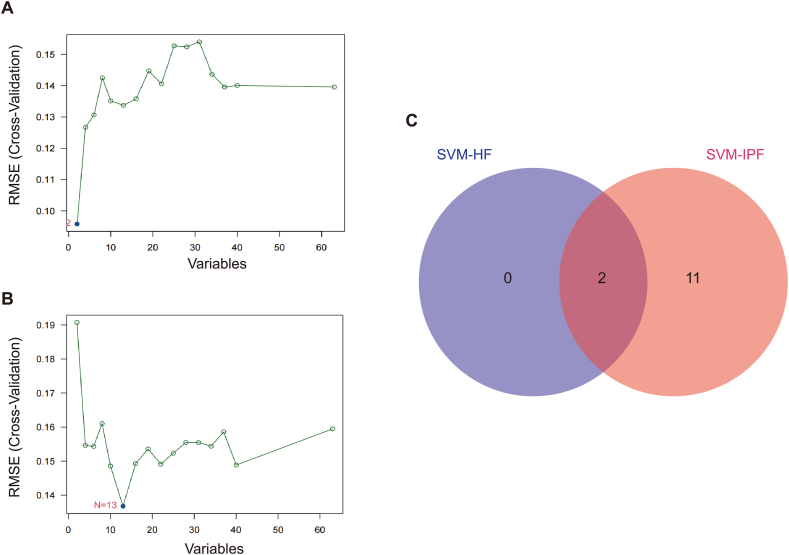
Identification of diagnostic markers in HF and IPF. (A) SVM-RFE algorithm to screen diagnostic markers in the GSE29819 database. (B) SVM-RFE algorithm to screen diagnostic markers in the GSE24206 database. (C) Venn diagram shows the optimal diagnostic biomarkers.

SVM-RFE is a machine learning technique that utilizes support vector machines to identify the most important gene by eliminating feature vectors generated by the SVM. Through this process, two genes (*CCND1* and *NAP1L3*) were identified as potential biomarkers (Fig. 6A–C). Therefore, these biomarkers may have diagnostic value.

### 3.5 Verification of shared diagnostic markers

**Fig. 7:**
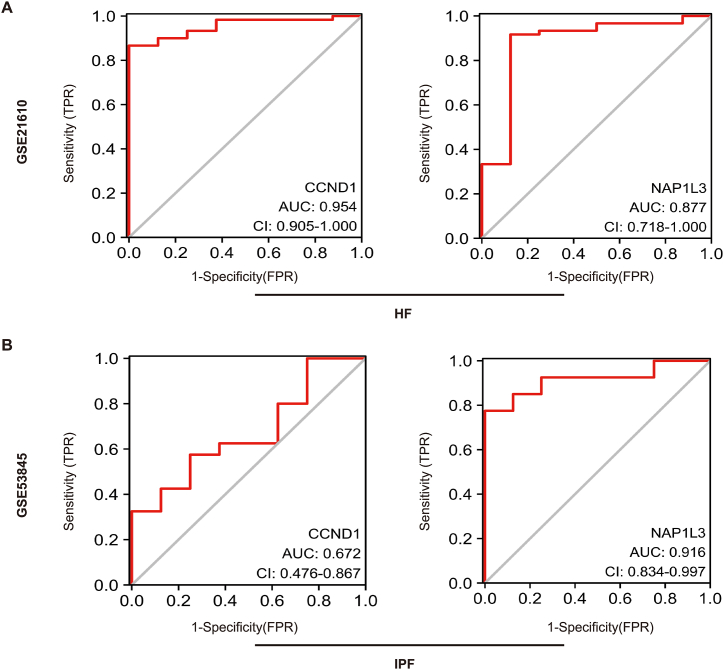
Validation of diagnostic shared biomarkers. (A) The ROC curve of the diagnostic efficacy verification in GSE21610. (B) The ROC curve of the diagnostic efficacy verification in GSE53845.

In addition, we verified the diagnostic effectiveness of these two indicators using external datasets. ROC curve analysis was conducted utilizing the GSE21610 and GSE53845 datasets. The GSE21610 dataset identified *CCND1* (AUC = 0.954) and *NAP1L3* (AUC = 0.877) as biomarkers with effective values for diagnosing HF, as shown in Fig. 7A. Similarly, the genes *CCND1* and *NAP1L3* showed robust predictive capacity for IPF (Fig. 7B). These findings validate the diagnostic significance of these two biomarkers in both diseases.

### 3.6 Immune infiltration analysis of shared biomarkers and diagnostic biomarkers

**Fig. 8:**
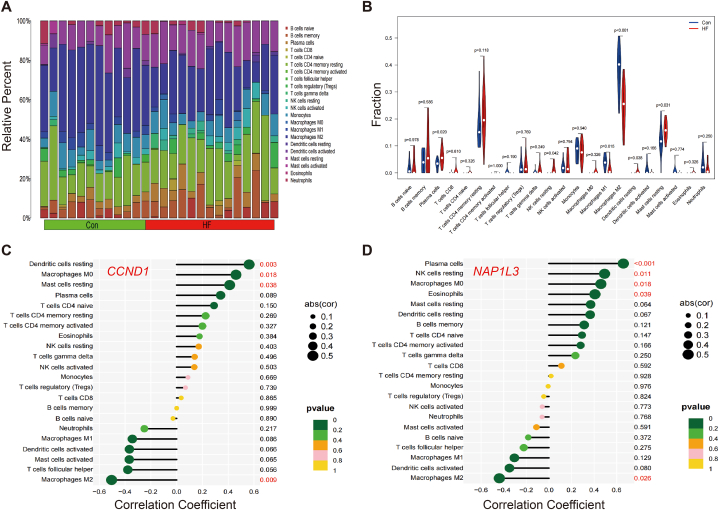
Immune infiltration analysis in HF. (A) The barplot of immune cell infiltration. (B) Violin diagram of the proportion of 22 types of immune cells. (C) In HF, correlation between *CCND1* and infiltrating immune cells. (D) In HF, correlation between *NAP1L3* and infiltrating immune cells.

**Fig. 9:**
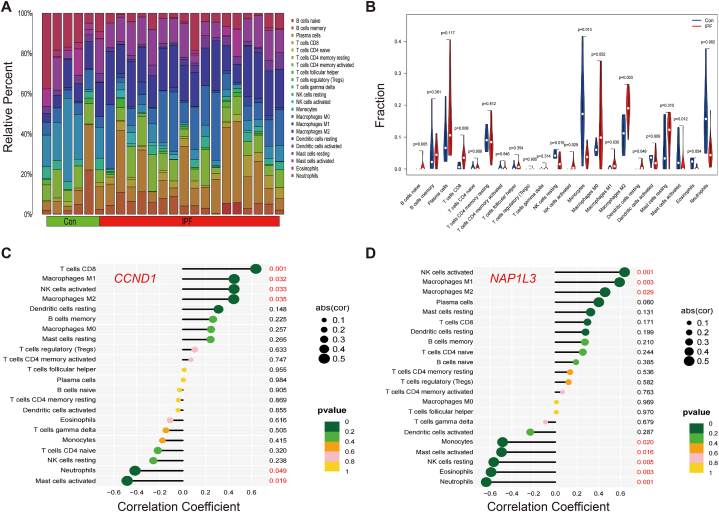
Immune infiltration analysis in IPF. (A) The barplot of immune cell infiltration. (B) Violin diagram of the proportion of 22 types of immune cells. (C) In IPF, correlation between *CCND1* and infiltrating immune cells. (D) In IPF, correlation between *NAP1L3* and infiltrating immune cells.

The 63 common genes' enrichment analysis revealed that immunity is crucial to the onset of HF and IPF. The CIBERSORT algorithm was employed to assess the prevalence of immune cells in various samples. The proportions of immunocytes in HF and IPF samples are shown in Fig. 8, Fig. 9A, respectively.

In comparison to the normal samples, the HF samples exhibited elevated levels of four types of immune cells, while M1 and macrophages M2 showed decreased levels (Fig. 8B). However, as compared to normal samples, IPF samples exhibited an increase in five types of immune cells, while four other types of immune cells showed a decrease (Fig. 9B).

Furthermore, the study investigated the relationship between diagnostic indicators and the composition of various immune cells. In the HF samples, there was a notable positive relationship between *CCND1* and resting dendritic cells, resting mast cells, and macrophages M0. Conversely, there was a substantial negative association between *CCND1* and macrophages M2 (Fig. 8C). In the case of *NAPlL3*, there was a notable positive relationship between *NAPlL3* and plasma cells, resting NK cells, M0 macrophages, and eosinophils. Additionally, there was a substantial negative association between *NAPlL3* and macrophages M2, as shown in Fig. 8D. In the IPF samples, there was a notable positive relationship between *CCND1* and CD8 T cells, activated NK cells, M1 and M2 macrophages. Conversely, there was a substantial negative link between *CCND1* neutrophils and active mast cells (Fig. 9C). In the case of *NAPlL3*, there was a notable positive relationship between *NAPlL3* and activated NK cells, M1 and M2 macrophages. Conversely, there was a substantial negative link between *NAPlL3* and resting NK cells, monocytes, active mast cells, neutrophils, and eosinophils (Fig. 9D).

The results of the immunoassay provided genetic evidence of immune cell invasion and immune mediated inflammation in the occurrence and development of HF and IPF, and indicated the immunological relevance and rationality of *CCND1* and *NAPlL3* as diagnostic biomarkers.

## 4 Discussion

In this study, we first identified 63 genes with a significant overlap between the HF- and IPF-related modules. The GO enrichment analysis revealed that these 63 common genes exhibited significant enrichment in several biological processes, including ECM and structural organization, neutrophil degranulation and immune response activation, as well as leukocyte movement implicated in inflammatory response. The KEGG analysis revealed that these shared genes were primarily linked to signaling pathways involved in protein digestion and absorption, ECM-receptor interactions, and focal adhesion.

The findings indicate that HF and IPF share significant signaling pathways, including ECM/structure organization, ECM-receptor interaction, focal adhesion, and protein digestion absorption. The findings we obtained align with the outcomes of prior researches conducted solely on HF and IPF. IPF is defined by the development of fibrous tissue and structural changes in the lungs, resulting from an abnormal healing response to damage of the alveolar epithelium [22]. HF is characterised by myocardial fibrosis and ventricular remodeling, mainly involving the repair of the myocardium after damage [1]. These fibrotic processes were closely related to various enriched pathways.

Moreover, a set of seven fundamental genes, including *COL1A1*, *COL3A1*, *THBS2*, *CCND1*, *ASPN*, *FAP*, and *S100A12*, have been recognized as central genes that play a crucial role in the common mechanisms of HF and IPF pathophysiology. The *COL1A1* gene expression product is the primary component of the ECM, and research has demonstrated its myocardial protective impact on HF [23]. Additionally, it has been recognized as a potential biomarker for the advancement of HF, specifically in predicting the progression from HF diagnosis to the survival rate one year after transplantation [23,24]. The COL3A1 gene encodes for the synthesis of the collagen alpha-1(III) chain. This protein is present in many fibrotic illnesses included multiple organs, such as heart, lung fibrosis, kidney and liver [[25], [26], [27]]. Collagen III contributes to cell adhesion, motility, and differentiation through its interaction with integrins [28] *THBS2* is associated with metastatic colorectal cancer, is shown to be expressed at higher levels in lung cancer, and participates in focal adhesion signalling pathways [[29], [30]]. *CCND1* gene regulates cell cycle progression and its upregulation promotes the development of various tumours. Asporin, encoded by *ASPN*, is a protein present in the ECM [[31], [32]]. *ASPN* affects the progression of keloids, pulmonary fibrosis, and cardiovascular fibrosis by hindering collagen metabolism [33]. The expression product of *FAP* is a integral membrane protein associated with fibrosis, tissue healing, and ECM degradation [[34], [35]]. *S100A12* is the gene responsible for producing a tiny protein that binds to calcium. This protein is involved in the inflammatory reactions triggered by neutrophils and monocytes/macrophages [36].

Through further induction and analysis of the above seven core genes, we found that *COL1A1*, *COL3A1*, *ASPN*, and *FAP* were mainly involved in the fibrotic and disordered collagen metabolism process, whereas overexpression of *THBS*2 and *CCND1* mainly promoted the cell cycle process and affected cell adhesion and migration. *S100A12* played a crucial role in the immunological response and facilitated the inflammatory response. The development of HF and IPF involves various pathological processes, including fibrosis, cell adhesion and migration, tissue repair, and immune pro-inflammatory responses. The seven screened core genes reflected and condensed the above processes. These hub genes have been reported separately in HF and IPF. Here, we screened and integrated for the first time and provided genetic evidence to reveal the common pathological basis of HF and IPF. TCF12, a transcription factor, interacted with multiple gene promoters and regulated biological processes such as embryonic development [37]. Five factors, particularly TCF12, were identified as significant transcription factors in the molecular regulation of these core genes, indicating that the pathogenesis of HF and IPF may involve similar upstream regulatory processes.

SVM-RFE was used to screen the feature genes. Two characteristic genes, *CCND1* and *NAP1L3*, were selected as diagnostic markers for HF and IPF, respectively, by using the SVM-RFE model. The verification results suggest that they are accurate. A recent study suggested that *CCND1* may serve as an underlying biomarker of idiopathic dilated cardiomyopathy [38]. In fact, idiopathic dilated cardiomyopathy is one of the most significant triggers of HF, and its histological manifestations include cardiomyocyte hypertrophy, interstitial fibrosis, and myofibril loss [24,39].

The findings of this investigation align with the outcomes of our machine-learning analysis. *NAP1L3* is a class of histone chaperone proteins that play a crucial role in various biological processes, including cell proliferation, transcriptional control, chromosome segregation, and DNA recombination and repair [40]. The results of our analysis indicated that the biological processes that were commonly shared primarily focused on the ECM and its structural organization, as well as neutrophil degranulation and activation. Similarly, the cell components that were commonly shared predominantly included the collagen-containing ECM and secretory granules. These processes involve the release of particles such as chemokines by inflammatory cells to trigger cell migration, which requires active substance synthesis and cell expansion [41]. This demonstrated the potential of *NAP1L3* as a predictor of HF and IPF.

Recent experimental and clinical studies have yielded fresh information that reinforces the significance of inflammation and immunity in the genesis and advancement of HF and IPF [[41], [42], [43]]. Neutrophils, mast cells, and macrophages are types of innate immune cells that have a crucial role in the development of cardiac hypertrophy and fibrosis through immune inflammation. Important substances involved in this immunoinflammatory process are IL-1β, IL-6, transforming growth factor (TGF)-β, and tumor necrosis factor (TNF)-α [44,45]. The number of inflammatory cells, including macrophages, antigen-activated T and B cells, mast cells, and lymphocytes, has been found to considerably increase in the circulation and fibrotic lungs of individuals with IPF. These findings align with the outcomes of our examination of immune cells in samples from patients with HF and IPF. In addition, we examined the pertinent infiltration characteristics of immune cells surrounding *CCND1* and *NAP1L3* in patients with HF and IPF. There was a strong positive connection between M0 macrophages and both *CCND1* and *NAPlL3* in the HF samples. There was a notable positive association observed between M1, M2 macrophages, and NK cells both *CCND1* and *NAPlL3* in the IPF samples. Monocyte-derived macrophages have the ability to release TGF-β, which has a pro-fibrotic effect by stimulating fibroblasts to change into myofibroblasts, resulting in the accumulation of collagen [46]. Nevertheless, NK cells are expected to have a defensive function in the formation of fibrosis, and the malfunctioning of NK cells is a significant characteristic of inflammatory and fibrotic disorders [47]. The correlation analysis findings indicate that *CCND1* and *NAP1L3* are valid biomolecular diagnostic indicators for HF and IPF.

Nevertheless, our study has three limitations. First, we utilized data extracted from a publicly accessible database, which had specific constraints pertaining to the size of the sample. Second, the efficiency of obtaining clinical information from public databases is limited. Third, there is insufficient evidence that *CCND1* and *NAP1L3* are powerful diagnostic markers for patients with HF and IPF. It is imperative that future experimental and clinical investigations are conducted to validate these findings.

The significant impact of HF and IPF on worldwide healthcare expenditures is concerning. Consequently, timely detection is crucial. This study employed bioinformatics techniques to identify genetic evidence elucidating the shared pathophysiological mechanisms of HF and IPF. The findings indicate that the pathogenesis of HF and IPF involves the ECM/structure organization, ECM-receptor interactions, protein digestion and absorption, and focal adhesion. In addition, we have discovered CCND1 and NAP1L3 as promising biomarkers for diagnosing HF and IPF. In brief, our findings offer novel genetic evidence for the co-pathogenesis of IPF and HF as well as potential molecular indicators for HF and IPF early diagnosis.

## Funding

This work was supported by the 10.13039/501100001809National Natural Science Foundation of China (81970289 and 82270340 to Junfeng Zhang; 82300362 to Tiantian Zhang), Experimental Animal Research Project of Shanghai Science and Technology Commission (22140901200 to Junfeng Zhang), Cross Research Fund Project of the Shanghai Ninth People's Hospital, 10.13039/501100004921Shanghai Jiao Tong University 10.13039/100008235School of Medicine (Special Project of 10.13039/501100009002Shanghai University of Science and Technology; JYJC202127 to Junfeng Zhang), and the SHIPM-mu fund from Shanghai Institute of Precision Medicine, Shanghai Ninth People's Hospital, 10.13039/501100004921Shanghai Jiao Tong University 10.13039/100008235School of Medicine (JC202005 to Tiantian Zhang).

## Ethics approval and consent to participate

Due to the public nature of the datasets used in this investigation, ethics approval and informed consent were not necessary.

## Data availability statement

Publicly available datasets were analysed in this study. These data can be found at https://www.ncbi.nlm.nih.gov/gds.

## CRediT authorship contribution statement

**Peng Zhang:** Writing – original draft, Methodology, Investigation, Formal analysis, Conceptualization. **Lou Geng:** Writing – original draft, Formal analysis, Data curation. **Kandi Zhang:** Writing – original draft, Investigation. **Dongsheng Liu:** Writing – original draft, Investigation. **Meng Wei:** Writing – original draft, Investigation. **Zheyi Jiang:** Writing – original draft, Investigation. **Yihua Lu:** Writing – original draft, Investigation. **Tiantian Zhang:** Writing – original draft, Investigation, Funding acquisition. **Jie Chen:** Writing – review & editing, Visualization, Validation, Investigation, Conceptualization. **Junfeng Zhang:** Writing – review & editing, Visualization, Validation, Investigation, Funding acquisition, Conceptualization.

## Declaration of competing interest

The authors declare that they have no known competing financial interests or personal relationships that could have appeared to influence the work reported in this paper.
